# Salivary Sialadenoma Papilliferum of Buccal Mucosa: A Case Report With Literature Review

**DOI:** 10.7759/cureus.95551

**Published:** 2025-10-27

**Authors:** Sridhar Reddy Padala, Munalisha Paul, Shambo Dey, Gaganjot Kaur, Bina Kashyap

**Affiliations:** 1 Oral and Maxillofacial Surgery, Agartala Government Dental College, Agartala, IND; 2 Oral Pathology and Microbiology, Agartala Government Dental College and Indira Gandhi Memorial (IGM) Hospital, Agartala, IND; 3 Oral and Maxillofacial Surgery, Agartala Government Dental College and Indira Gandhi Memorial (IGM) Hospital, Agartala, IND; 4 Oral Pathology, Shaheed Kartar Singh Sarabha Dental College and Hospital, Ludhiana, IND; 5 Oral Pathology, Agartala Government Dental College, Agartala, IND

**Keywords:** benign, malignant, microscopy, salivary gland, sialadenoma papilliferum

## Abstract

Salivary sialadenoma papilliferum (SP), an infrequent benign salivary gland neoplasm, resembles an uncommon benign tumor of sweat gland origin called syringocystadenoma papilliferum. The purpose of this paper is to report a case of SP of a minor salivary gland, near the parotid gland orifice, in an adult female patient presenting as intraoral growth and extraoral swelling. The present case highlights the SP's clinical and microscopic features, considering other exophytic papilliferous oral lesions and understanding the need to explore the nature and malignant potential of the tumor.

## Introduction

Salivary sialadenoma papilliferum (SP) is a rare benign neoplasm that tends to affect the minor than the major salivary glands of the oral cavity. It belongs to papillary salivary gland tumors, consisting mainly of inverted ductal and intraductal papilloma. Other papillary salivary gland tumors include exophytic ductal papilloma and papillary cystadenoma [1]. The World Health Organization (WHO), in its 2017 classification, separated the three papillary salivary gland tumors into two: (i) SP and (ii) ductal papilloma (intraductal and inverted type) [2]. In 1969, SP was first described by Abrams and Finck, wherein SP's resemblance to a benign tumor of the sweat gland was highlighted [3]. According to the Armed Forces Institute of Pathology, SP comprises 0.4% of all minor salivary gland neoplasms [4]. SP is a painless exophytic papillary mass affecting life's fifth to seventh decades with a male predilection. Its characteristic biphasic histological growth pattern is observed mainly in the hard palate. However, it is also observed in the soft palate, buccal mucosa, upper lip, and nasal cavity. SP's rare appearance is also reported in the bronchus and esophagus [5].

The characteristic clinical features of SP are a solitary exophytic lesion appearing white, round, asymptomatic, with limited growth within a diameter of 1-2 cm. The slow-growing papillary projections easily lead to the clinical impression of squamous papilloma. However, no association has been found between SP and oral human papillomavirus infection. Furthermore, there are other similar appearing clinical papillary lesions in the oral cavity, such as verruca vulgaris, verruciform xanthoma, verrucous carcinoma, or papillary squamous cell carcinoma [6]. SP is a non-encapsulated benign tumor presenting as an exophytic and endophytic oral mucosal growth that consists of salivary ducts and mucosal epithelium microscopically. The nature of SP is debatable, particularly its squamous component. The excretory duct or its reserve cells are speculated to contribute to SPs' exophytic and endophytic components. Most of the reported SP cases are benign and are treated successfully by complete excision. Despite SP having a good prognosis, malignant transformation in pre-existing SP has been reported but not conclusively proven [4]. Herein, a case report of SP occurring on the parotid gland duct orifice is presented, as a sessile exophytic mass intraorally and extraoral swelling in the cheek, which confused the clinical diagnosis. We highlight the unique presentation of the case compared to other reported cases in the literature and emphasize its inclusion with other peripheral lesions. Also, the current advancements in diagnosing SP and the differential points that should be considered during diagnosis are discussed.

## Case presentation

**Figure 1:**
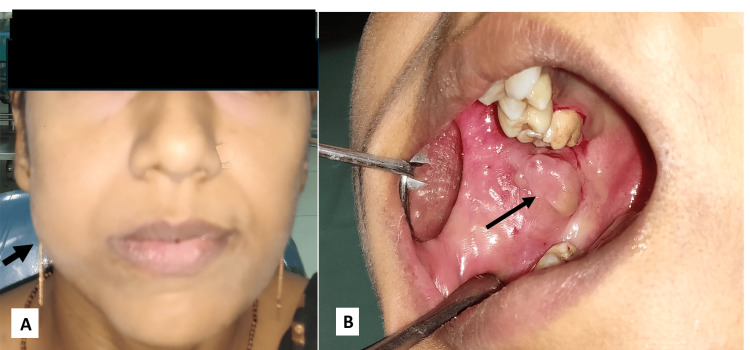
Clinical presentation of the patient. Extraoral swelling in the right cheek (A, black arrow). Intraoral lobulated, painless, sessile soft tissue growth near the parotid orifice (B, black arrow). Image credit: Sridhar Reddy Padala

A 30-year-old woman visited the outpatient clinic complaining of painless, growing swelling in the right cheek for two years. Extraoral examination revealed diffuse swelling on the right side of the cheek (Figure 1A). Intraoral examination showed around 3 to 2 cm exophytic soft fibrous mass near the orifice of the parotid duct on the right buccal mucosa. The lesion was slightly lobulated, pale pink, with a smooth and shiny superficial surface. No erythema, ulceration, or tenderness was observed (Figure 1B). According to the patient, there had been no pain. No past medical or dental histories were specified by the patient. Clinical diagnosis of either lipoma or fibroma was established. Other dental findings revealed a few carious teeth and generalized gingivitis, for which the treatment plan was explained to the patient. The patient admitted to having paan without areca nut weekly once for the past six years. An excision biopsy of the lesion was planned, and informed consent was obtained before treatment. The whole tissue was excised in toto. The excised specimen was submitted for histopathological analysis as an exophytic mass with an underlying tumor base. At the time of suture removal, the patient's extraoral swelling had subsided. 

**Figure 2:**
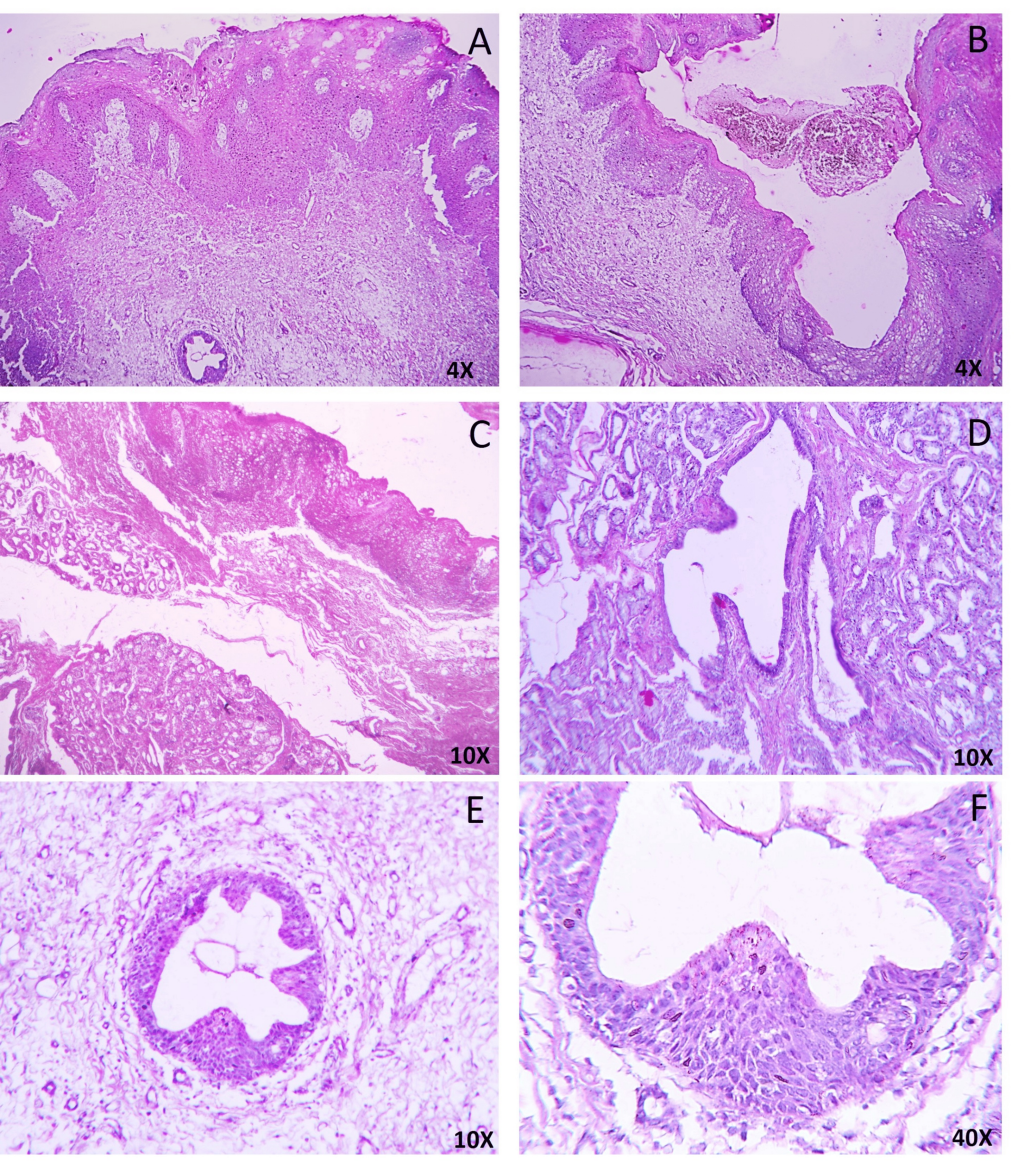
Histological photomicrograph showing a biphasic growth pattern of sialadenoma papilliferum (SP). Exophytic mass with papillary proliferations lined by keratinized stratified squamous epithelium (A, 4X). Presence of cleft at the point of fusion of the surface epithelium (B, 4X). Numerous minor mucous salivary glands with extralobular dilated tortuous ducts in the connective tissue (C and D, 10X). Low-power view of the papillary infoldings of the glandular ductal structures (E, 10X) and the bilayered/multilayered ductal structures lining with a flattened basal cell layer and a cuboidal-to-columnar luminal layer, at high power (F, 40X). Image credit: Bina Kashyap

Histopathological examination revealed distinct pathological appearances. The exophytic growth showed papillary proliferations lined by keratinized oral epithelium without nuclear atypia (Figure 2A). The papillae are supported by fibrovascular cores showing inflammatory cell infiltration, including plasma cells and lymphocytes. The epithelium also showed cleft and endophytic proliferation of glandular structures in the connective tissue (Figure 2B). The connective tissue beneath the papillary proliferations presented numerous minor mucous salivary glands with extralobular dilated tortuous ducts (Figures 2C, 2D). The ducts are bilayered/multilayered, showing cuboidal/columnar cells or cuboidal/basal cells lining (Figures 2E, 2F). Based on the above features, the diagnosis of SP was established. As the lesion was excised adequately, no additional therapy was advised. Considering the nature and literature regarding SP, the patient has been on regular follow-ups for the past year (12 months), and no recurrence has been observed.

## Discussion

SP is a rare salivary gland condition, the clinical presentation of which is confused with other lesions occurring in the oral cavity, such as fibroma, squamous papilloma, and mucocele [6]. They present as small exophytic asymptomatic nodules, which makes it impossible to conclude a diagnosis only with clinical information. Our case uniquely presented an extraoral swelling and an intraoral nodular growth in a female patient in her third decade of life. Due to its small size, clinical findings of extraoral swelling have not been reported in the English-language literature. The extension of the lesion in the present case confronts the SP growth pattern.

From the time SP was described, its histogenesis remained controversial and is still not fully understood. The authors who first described this lesion believed it to be of myoepithelial origin because the tumor cells revealed the immunoreactivity for smooth muscle actin [3]. Later, the intercalated duct cell origin was reported because tumor cells co-expressed cytokeratin, vimentin, and desmin [7]. SP originating from the excretory duct cell was proposed for its capability of multidirectional differentiation [1]. Conversely, others have proposed that SP is a result of focal hyperplasia after salivary gland duct obstruction [4]. Recently, the genetic mutations of BRAF and HRAS have been reported in SP, considering it to be the salivary complement of benign sweat tumor of skin, syringocystadenoma papilliferum. Also, SOX10 immunopositivity of ductal cells suggested SP origin from intercalated duct or ductal progenitor cells [5,8]. With the complex histopathological, immunohistochemical, and molecular features of SP, it is speculated that tumor cells are derived from several glandular components. BRAF analysis and SOX10 immunostaining are useful for a positive diagnosis. Despite the controversies regarding the cell of origin, the SPs microscopically favor their derivation from excretory duct reserve cells. These reserve cells have the potential to differentiate into both a ductal and squamous cell type.

Histopathological examination is the gold standard to confirm an SP diagnosis and to rule out the possibility of other similar lesions (Table 1). Two histological variants of SP have been reported, namely classic and oncocytic variants. Classic SP has bilayered or multilayered structures, a papillary squamous surface, and an endophytic part of ductal structures composed of columnar or cuboidal cells, whereas in oncocytic SP, the papillary endophytic ductal component is composed of oncocytic cells that merge with the stratified epithelium [8]. The absence of oncocytic cells and the features of classic SP are evident in the present case. Though the buccal mucosa is the second most affected site after the palate, very few cases are reported. Considering the limited description of SP occurring in the buccal mucosa, the malignant transformed cases reported so far are presented in Table 2 [7-17]. Nearly 95 cases of SP are reported in English language literature in the Head and Neck region, and only five cases of SP are reported with malignant transformation. As very few malignancies are reported with SP, we believe there are insufficient publications to comment on whether the malignancies originated from a pre-existing SP. Therefore, this tumor entity virtually has no malignant potential. On the contrary, pathologists must be aware of the possibility of malignant change in this rare lesion.

**Table 1:** Differentiating points of sialadenoma papilliferum from other papillary salivary gland tumors.

Salivary gland tumors	Gross appearance	Exophytic papillary feature	Lining epithelium	Submucosa component
Sialadenoma papilliferum	Papillary and exophytic	Present–surface squamous cells with clefts	Bilayer- lined by luminal cuboidal to columnar cells and cuboidal to flattened basal cells	Small duct-like structures, papillary infoldings, merge with the mucosal surface, forming a cleft
Exophytic ductal papilloma	Papillary and exophytic	Present – squamous, ductal, and transitional cells	Cuboidal or columnar cells and transitional cells	Single ectatic duct showing papillary proliferation, near the ductal orifice
Inverted papilloma	Submucosal mass/nodule	Absent	Squamous, transitional, and columnar cells	Endophytic papillary proliferation, excretory duct involvement, occasionally attached with mucosal surface
Intraductal papilloma	Submucosal mass/nodule	Absent	Cuboidal or columnar cells	Papillary proliferations into the lumen
Papillary cystadenoma	Submucosal mass/nodule	Absent	Cuboidal or columnar cells	Multiple ductal and cystic structures of variable sizes

**Table 2:** A detailed description of sialadenoma papilliferum occurring in the buccal mucosa and malignant transformed cases. BM: Buccal mucosa

S.No	Age (Years) /Sex	Location	Duration/Size(cm)	Clinical appearance	Histopathological diagnosis	Follow-up	Reference
1	2Y/M	BM	NA/0.4	NA	Classic SP	NA	[8]
2	61Y/M	BM	NA/0.2	NA	Oncocytic SP	NA	[8]
3	67Y/F	BM	12 months/2	Sessile mass	Classic SP	Recurrence - 36 months	[9]
4	60Y/M	BM	NA/0.8	Squamous papilloma	Classic SP	NAD - 96 months	[7]
5	77Y/M	BM	NA	NA	Classic SP	NA	[10]
6	53Y/M	BM	5 months/0.8	Accidental finding, Painless, exophytic mass	Classic SP	NAD – 24 months	[11]
7	77Y/F	BM	NA/0.7	Papillary growth indurated	Classic SP	NAD	[12]
8	75Y/M	BM	NA/0.6	NA	Classic SP	NA	[8]
Malignant transformation of SP reported							
1	62Y/M	Soft palate		Exophytic papillary mass	Associated malignancy – Squamous cell carcinoma	Multiple recurrences	[13]
2	79Y/F	Junction (hard and soft palate)		Exophytic pink-white papillary mass	Epithelial-myoepithelial carcinoma	Expired	[14]
3	30Y/M	Floor of mouth		Exophytic, slightly papillary lesion	Carcinoma in situ	NAD – 8 months	[15]
4	82Y/F	Base of tongue		Exophytic papillary mass	Mucoepidermoid carcinoma	NA	[16]
5	67Y/F	Retromolar trigone		Warty-surfaced large mass	Dysplasia	NAD - 3Y	[17]

SP is not encapsulated microscopically but has a favorable prognosis with conservative surgical removal, and in most cases, no further treatment is required. The present case was excised considering a clinical diagnosis of lipoma, and care was taken to remove the complete tissue with extra margins. Also, in our case, the typical histological features of SP made us exclude any further molecular testing. Recently, trans-oral robotic surgery has been proposed for the successful removal of SP tumors. It is a novel technique used for head and neck surgery [18].

## Conclusions

In the present case, histopathological diagnosis has led to exploring SP, which was unnoticed by clinicians and surgeons, hence emphasizing including it in the clinical diagnosis of peripheral lesions of the buccal mucosa. To summarize, clinical and histologic diagnoses require caution when dealing with papillary lesions occurring at the salivary gland orifice. Immunohistochemistry or molecular testing may be useful diagnostic adjuncts to confront challenges with intraoral salivary gland neoplasm. Also, more documentation of malignant SP in the literature would support explaining the biological behavior and improve management.
